# Novel ZnO:Al contacts to CdZnTe for X- and gamma-ray detectors

**DOI:** 10.1038/srep26384

**Published:** 2016-05-24

**Authors:** U. N. Roy, R. M. Mundle, G. S. Camarda, Y. Cui, R. Gul, A. Hossain, G. Yang, A. K. Pradhan, R. B. James

**Affiliations:** 1 Brookhaven National Laboratory, Upton, NY 11973, United States; 2 Department of Engineering, Norfolk State University, Norfolk, VA 23504, United States

## Abstract

CdZnTe (CZT) has made a significant impact as a material for room-temperature nuclear-radiation detectors due to its potential impact in applications related to nonproliferation, homeland security, medical imaging, and gamma-ray telescopes. In all such applications, common metals, such as gold, platinum and indium, have been used as electrodes for fabricating the detectors. Because of the large mismatch in the thermal-expansion coefficient between the metal contacts and CZT, the contacts can undergo stress and mechanical degradation, which is the main cause for device instability over the long term. Here, we report for the first time on our use of Al-doped ZnO as the preferred electrode for such detectors. The material was selected because of its better contact properties compared to those of the metals commonly used today. Comparisons were conducted for the detector properties using different contacts, and improvements in the performances of ZnO:Al-coated detectors are described in this paper. These studies show that Al:ZnO contacts to CZT radiation detectors offer the potential of becoming a transformative replacement for the common metallic contacts due to the dramatic improvements in the performance of detectors and improved long-term stability.

X-ray and gamma-ray detectors are an essential element today in several astronomical telescopes, and they are invaluable for various field applications in non-proliferation, national security, and medical imaging. CdTe and CdZnTe (CZT) crystals are the leading materials for room-temperature semiconductor radiation detectors designed to meet the needs for many such uses^1^,^2^,^3^,^4^,^5^,^6^. Long-term stability is highly important for most of these field applications. However, one critical challenge facing this technology is the instability of detectors over time due to the sub-optimal interconnection between the metal contacts and the semiconductor detecting material. This interfacial layer between the contact and crystal plays a vital role in the detector’s performance^7^, and much of the premature degradation of the device’s performance is caused by the poor adhesion of the contact and crystal. The main reason for the contact’s poor adhesion is the large difference in the thermal-expansion coefficients of the common metals used for contacts and the underlying CZT crystal^8^. Because of this difference, a large stress inevitably exists at the contact/semiconductor interface due to changes in the ambient temperature, such as when the crystal is cooled from higher temperatures to room temperature during and immediately following the deposition process. This stress between the metal and the CZT causes mechanical degradation of the electrode, and so it adversely affects the stability of the contact/semiconductor interface and the overall performance of the detector. Furthermore, often the contacts formed by the conventional processes used today peel off from the CZT crystal over time, especially for electron beam and thermally deposited gold and platinum electrodes. Sang *et al*.^8^ showed that at the beginning stages of accelerated aging studies, the interfacial adhesion decreases drastically, which causes degradation in the detector performance. The problem of interfacial adhesion has been shown to exist for most of the common metals, such as Au, Al, In and Pt, that are used for CdTe/CZT^8^,^9^ devices. Although these conventional metallic contacts are being used widely for commercial nuclear detectors based on CdTe and CdZnTe, their long-term reliability remains a concern.

To resolve this problem in nuclear-radiation detector applications, we adopted a transparent conductive oxide (TCO) to replace the commonly used metallic contacts. These transparent conductive oxides essentially are degenerate semiconductors, wherein the Fermi level (*E*_*f*_) lies within/above the conduction band (*E*_*c*_), as is the case for metals. These heavily doped degenerate semiconductors possess more metallic than semiconductor properties concerning their electrical resistivity. In this research, we chose aluminum-doped ZnO (AZO) for applying the contact to CdZnTe for nuclear-detector applications. Doped (Al) ZnO-based TCO can be grown with a resistivity as low as ~8 × 10^−4^ ohm-cm^10^. Furthermore, AZO offers several advantages over the conventional metallic contacts that now are used for radiation detectors. Owing to the oxide nature of the contact, the interface assures better chemical adhesion to the CdZnTe surface and improved ambient stability of the electrode/CdZnTe interface. The thermal-expansion coefficient of ZnO closely matches that of CdZnTe, so reducing the mechanical stress at the interface. In addition, the hardness of ZnO is 8–20 times higher than that of conventional metals. We demonstrated that the AZO contact to CdZnTe nuclear-radiation detectors performs better compared to a device fabricated from a conventional metal contact.

## Results

**Figure 1:**
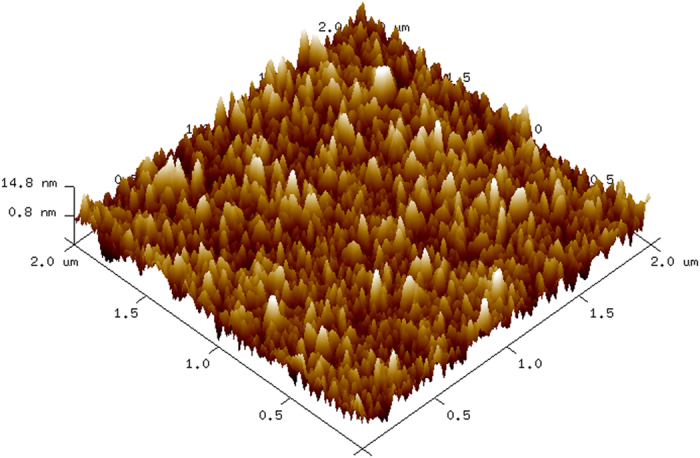
Atomic force microscopic image of an ALD-grown AZO film deposited on a CZT surface.

We deposited AZO thin films on the surfaces of Cd_0.9_Zn_0.1_Te detectors by an atomic layer deposition (ALD) technique. The Zn:Al composition ratios were grown via the alternate deposition of diethyl zinc (DEZ, Zn(C_2_H_5_)_2_), H_2_O and trimethyl-aluminum (TMA, Al(CH_3_)_3_). The details of the deposition process are reported elsewhere^10^. In the present case, the Zn:Al ALD cycle ratio was maintained at 30:1, and the corresponding measured Al atomic concentration was ~3% (Fig. 1 of the supplementary information in Ref. 10). The CZT-metal contact is known to degrade at ~100 °C and above^11^,^12^ causing increased leakage current of the detector and deterioration of its performance. In order to increase the throughput of fabricating high-quality detectors, we performed the AZO deposition process at a moderately low temperature of 85 °C. The measured resistivity of the deposited AZO film, 250-nm thick, was 3.8 × 10^−2^ ohm-cm, which is a little higher than that reported earlier^10^, perhaps due to the low-temperature deposition process. Figure 1 depicts a 2 × 2 μm^2^ scan area of the AZO film surface morphology using atomic force microscopic (AFM). It shows the small-grain polycrystalline nature of the film, which is typical for ALD-deposited AZO films^13^. Commercial good-quality detector-grade Cd_0.9_Zn_0.1_Te crystals were used for fabricating the radiation detectors. For the present study, we used the virtual Frisch-grid geometry for the detector configuration.

**Figure 2:**
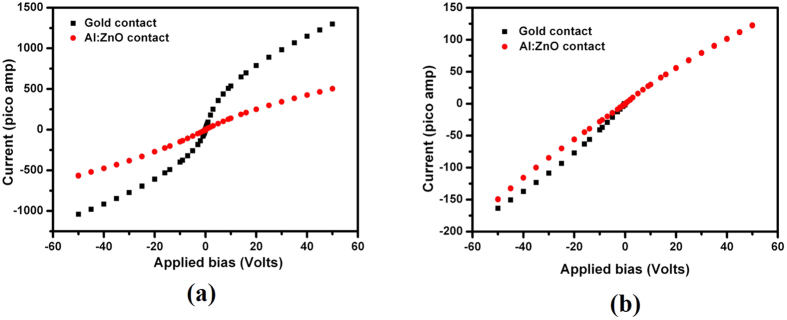
Current-voltage characteristics at room temperature of Frisch-grid detectors with gold and AZO contacts on the same sample, (**a**) detector 1 and (**b**) detector 2.

The virtual Frisch-grid geometry is designed for single-charge-carrier (electron) type sensors; it is used for producing very high resolution detectors for X- and gamma-ray applications^14^,^15^,^16^,^17^. They essentially are bar detectors, and for the present demonstration, we chose a standard dimension for the bar detectors of ~5 × 5 × 14 mm^3^ and ~6 × 6 × 15 mm^3^. The contacts were deposited on 5 × 5 mm^2^ and 6 × 6 mm^2^ faces, and the side walls were covered with a heat-shrink thin insulating polyester tube onto which the aluminum Frisch grid was wrapped. For comparison, detectors were fabricated with gold-contact electrodes deposited from gold-chloride solution, and the same detectors were re-fabricated with AZO-contact electrodes, so that comparative assessments of the detectors’ performance could be carried out under the same conditions. Cu-Be spring contacts were used for electrical contacts to both the cathodes and the anodes. We described details of the fabrication of the detectors elsewhere^16^. The schematic of the Frisch-grid detectors with a gold contact and AZO contact are shown in Fig. 1 of the supplementary information. In the present study, we demonstrated the performance of two detectors with ohmic contacts and back-to-back Schottky type contacts, which are typical for CZT/CdTe detectors. The room temperature current-voltage characteristics of the same detector, with a gold and with AZO contact, are shown in Fig. 2. Figure 2a shows the typical Schottky type current-voltage characteristics for the ~5 × 5 × 14 mm^3^ sample (detector 1), while the ohmic nature of the current-voltage characteristics for the ~6 × 6 × 15 mm^3^ sample (detector 2) is shown in Fig. 2b. For both I-V characteristics, the dark current with the AZO contact was found to be lower compared to that with a gold contact. The lower dark current is highly desirable for nuclear radiation detectors, since it enhances the signal-to-noise ratio of the device.

**Figure 3:**
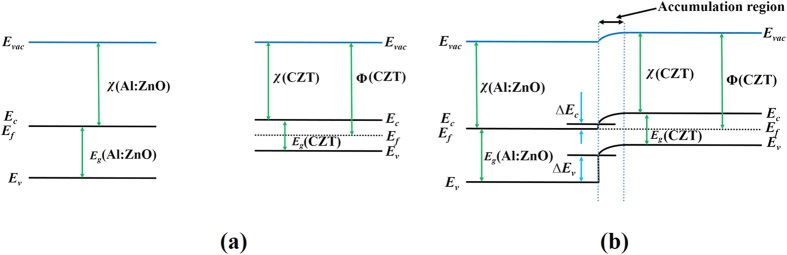
(**a**) Flat-band energy diagram of Al:ZnO and CZT before they are brought into contact, and (**b**) band diagram of AZO/CZT at thermal equilibrium.

The added advantage of lower dark leakage current is that it increases the possibility of operating devices at higher applied-bias voltages, as it is well known that the devices offer a heightened charge collection with improved resolution at higher applied bias voltages. We found that the nature of the current-voltage characteristic is more ohmic with the AZO contact layer than with a gold contact (Fig. 2a). This can assure more uniform distribution of the electric field inside the detectors, and it is advantageous especially for long drift-length (thicker) detectors. Figure 3 is a schematic of the energy-band diagram of the new AZO-CZT structure. Figure 3a,b, respectively, show the flat-band energy diagram of Al:ZnO and CZT before they are brought into contact and the band diagram of AZO/CZT at thermal equilibrium, where *E*_*vac*_, *E*_*f*_, *E*_*c,*_ and *E*_*v*_, respectively, are the vacuum energy, Fermi energy, conduction, and valence-band energy levels. χ is the electron affinity, and Φ is the work function. The band-gap energy is defined by *E*_*g*_. In the case of Al:ZnO, replacing the group II element, Zn, by group III element Al produces more free electrons and makes ZnO more and more n-type as the level of Fermi energy approaches the conduction band. For heavily Al-doped ZnO, the Fermi level moves within/above the conduction band minimum making ZnO a degenerate semiconductor. In this case, the electron affinity, χ, and the work function, Φ, essentially become the same as for heavily doped Al:ZnO, as illustrated in Fig. 3a. The value of χ (Al:ZnO) is 4.35 eV, and the band gap of ZnO is 3.3 eV^18^. In contrast to heavily doped Al:ZnO, for radiation-detector applications, very high resistivity semi-insulating Cd_0.9_Zn_0.1_Te with resistivity >2 × 10^10^ ohm-cm is necessary to operate these devices under a very high applied bias^7^,^14^,^15^,^16^,^17^. The flat-band energy diagram of semi-insulating CZT is shown in Fig. 3b. In order to estimate the work function of CZT (Φ(CZT)), the band gap of Cd_0.9_Zn_0.1_Te is necessary. However, the reported band-gap of Cd_0.9_Zn_0.1_Te varies widely over the range of 1.51–1.6-eV at room temperature (ref. 19 and the references therein). The band gap of Cd_1−x_Zn_x_Te can be calculated from the following empirical formula reported by Prokesch *et al*.^20^


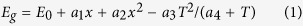


where *E*_*0*_ = 1.606 eV, and the values for *a*_*1*_, *a*_*2*_, *a*_*3*_, and *a*_*4*_are 0.38 eV, 0.463 eV, 4.5 × 10^−4^ eV/K and 264 K respectively^20^. T is the temperature in K, and *x* is the atomic composition. The calculated band-gap for Cd_0.9_Zn_0.1_Te at 300 K is *E_g_* = 1.577 eV.


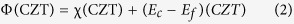


The value of χ (CdTe) is ~4.3 eV^21^, and since the CZT used for radiation detector applications is semi-insulating, we can assume that the Fermi energy level lies near the middle of the band gap^21^, thus (*E_c_ − E*_*f*_) ≈ *E_g_*/2 = 0.79 eV. Then, Φ(CZT) = (4.3 + 0.79) eV = 5.09 eV. The value for χ(Al:ZnO)  = 4.35 eV. Hence, in the present case χ(Al:ZnO) < Φ(CZT), implying that, upon contact of AZO to CZT, AZO will inject electrons to level off the Fermi energies, and the conduction band of CZT will bend down towards the CZT Fermi level. Figure 3c depicts the ideal energy band diagram of the AZO-CZT contact in thermal equilibrium. The ∆*E_c_* and ∆*E_v_* are the conduction- and valence-band offset, respectively. However, the actual energy-band diagram might differ drastically due to the presence of high densities of defects at the interface caused by surface damage and the large lattice mismatch between ZnO and CdTe/CZT. It is to be noted that the work function of gold (Φ_M_) is 5.1–5.47^21^. In the present case Φ_M_ > Φ(CZT) while χ(Al:ZnO) < Φ(CZT), thus, if a gold contact creates a Schottky contact for CZT, Al:ZnO is expected to be ohmic in nature for the same CZT and vice versa. However, as shown in Fig. 2a,b, the I-V characteristics show similar behavior for both gold and Al:ZnO contact on the same CZT surface. This depicts the fact that the band bending for the AZO/CZT contact is predominantly governed by the interface density of states rather than the work function differences. The presence of a high concentration of interface defect states significantly influences the band bending, conduction, and valence-band offsets. The calculated density of defect states at the interface of ZnO/CdTe was reported^18^ to be as high as 4 × 10^14^ cm^−2^. A large concentration of interface defect states generally is observed also at the metal/CZT interface^20^. In the present study, the better ohmicity of our AZO/CZT contact compared to that of gold/CZT contact with a lower dark current offers a profound advantage in improving the uniformity of the internal electric field and increasing the charge collection.

**Figure 4:**
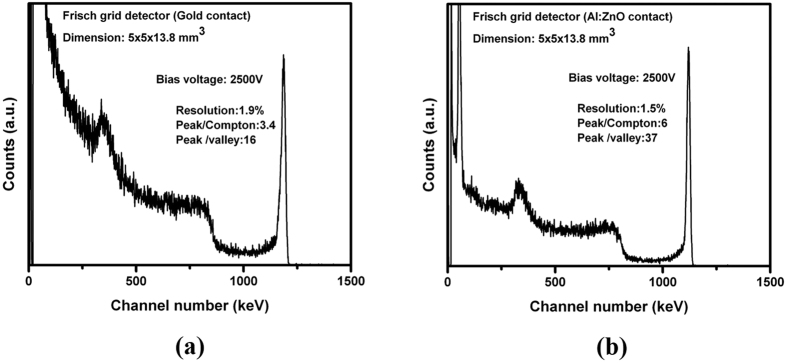
Pulse height spectra from ^137^Cs gamma source for the virtual Frisch-grid detector #1, (**a**) with gold contacts and (**b**) with AZO contacts.

The detector’s response was evaluated at room temperature. The representative pulse-height spectra for the detector (detector 1) fabricated with gold electrodes on both the cathode and anode sides is shown in Fig. 4a, while Fig. 4b shows the pulse-height spectrum of the same detector re-fabricated with AZO contacts on both the cathode and anode sides. Both the spectra were acquired from a ^137^Cs source placed ~3 mm away from the sample on the cathode side. Both spectra were acquired under an applied bias of 2500 V across the anode keeping the same shaping time. The spectra show an intense peak corresponding to the 662-keV line of the ^137^Cs source. As Fig. 4 shows, the performance of the detector with the AZO contact significantly improved compared to the same crystal with Au contacts. The detector’s resolution improved from 1.9% to 1.5% after replacing the gold with the AZO contact. In addition, the peak-to-Compton ratio and the peak-to-valley ratio, which are important figures of merit for CZT detectors, were found to be superior for the AZO/CZT contact detector compared to the gold contact, as illustrated in Fig. 4.

**Figure 5:**
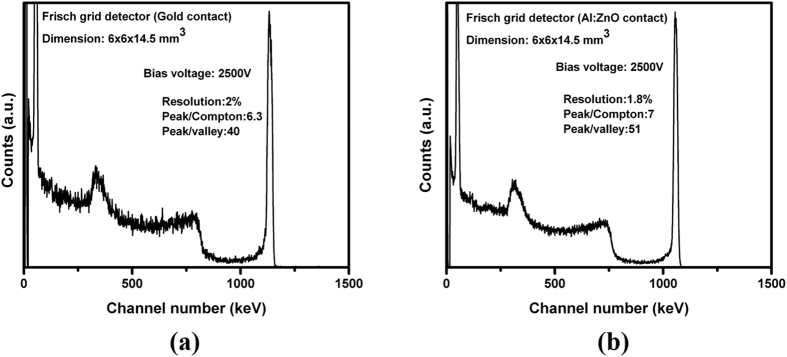
Pulse height spectra from a ^137^Cs gamma source for the virtual Frisch-grid detector #2, (**a**) with gold contacts and (**b**) with AZO contacts.

The detector response of the other detector (detector 2), which showed ohmic behavior was also evaluated and compared for gold and AZO contacts. The pulse height spectra of the same detector fabricated with gold and AZO contacts for the ^137^Cs source acquired at room temperature are shown in Fig. 5a,b, respectively. The resolution, the peak-to-Compton ratio, and the peak-to-valley ratio for the 662-keV line were also noted to be improved with AZO contacts as compared to gold contacts. It is thus evident that for both type of detectors (Schottky and ohmic), the detector performance is enhanced for AZO contacts compared to standard gold contacts. However, for Schottky type detectors the performance enhancement is more pronounced, due to the more ohmic nature of the AZO contacts with respect to gold contacts, as shown in Fig. 2a.

In addition to enhanced sensitivity and improved detector response, AZO contacts offer more advantages over conventional metallic contacts in regard to long-term stability. As discussed earlier, most of the common metals adhere poorly to CZT, and moreover, the large mismatch between the thermal-expansion coefficient of CZT and the commonly used metals exerts large stress at the metal/CZT interface. This stress between them causes mechanical degradation and thus adversely affects the detector’s performance and rate of aging. Table 1 (see supplementary information). lists the thermal-expansion coefficients of the common metals and CZT. Compared to the common metallic contacts, the thermal expansion coefficient of ZnO is the closest match to the thermal expansion coefficient of CZT (Table 1 in supplementary information). Thus, the AZO/CZT interface will experience less thermal stress compared to the commonly used metallic contacts, and hence offers devices better mechanical stability resulting in improved long-term stability. The hardness of ZnO is about 8–20 times higher (Table 1 of supplementary information) compared to the commonly used contact metals. Because of this, the ZnO contact is more rugged and relatively scratch proof during processing and handling as opposed to the metal contacts, especially for bump-bonding contacts for pixelated detectors. The AZO was found to have better chemical adhesion to CZT surfaces in addition to its hardness. The metal contacts can be easily polished off the surface of CZT with alumina suspension on a soft felt pad, while AZO contacts had to be lapped on SiC lapping paper confirming the improved adhesion and greater hardness of AZO contact to CZT. Our low-temperature photoluminescence (PL) study also revealed improved ambient stability and better interface (see the PL results Supplementary information).

The present experimental results clearly demonstrate the role of AZO as a promising alternative contact-layer to the common metals for CdTe-based nuclear-radiation detectors operating at room temperature. In addition to the several advantages of ZnO, such as higher hardness, better chemical stability of the interface, close match of thermal expansion coefficient of CdTe/CdZnTe and ZnO, the detectors with AZO contacts demonstrated better sensitivity and improved spectral performance. Thus far, our AZO/CZT detectors have been working for eleven months without any signs of degradation in their performance. Their enhanced performance might be due to the more ohmic/injecting nature of the contacts with a lower dark current, thereby resulting in better charge collection and lower noise. Further analyses are being carried out to gain more insights into the details of the mechanism behind these novel findings.

## Methods

We deposited aluminum-doped ZnO films on CdZnTe samples using a Cambridge Nanotech Savannah 100 atomic layer deposition (ALD) system. The Zn:Al composition ratios were controlled via alternate depositions of diethyl zinc (DEZ, Zn(C_2_H_5_)_2_), H_2_O, and trimethyl-aluminum (TMA, Al(CH_3_)_3_). The details of the deposition process are reported elsewhere^10^. In the present case, the Zn:Al ALD cycle ratio was maintained at 30:1. The temperature of the chamber was maintained at 85 °C during the entire deposition process. The thickness of the deposited AZO layer was 250 nm. Detectors were fabricated with gold and with AZO contacts; both types of contacts were deposited on polished surfaces. The CZT samples were polished to a mirror-like finish by final polishing with a 0.1-μm alumina suspension on a felt pad. The gold contacts were deposited from a gold chloride solution on the cathode and anode sides. The same detectors were later re-fabricated by polishing off the gold contacts and depositing AZO on the freshly polished surface of the CZT on the cathode and anode sides. The side walls of the detectors were insulated by a thin polyester heat-shrink tube. The CZT detector was placed inside the polyester tube and plugged at both open ends by an aluminum bar and immersed in water of 85–90 °C, whereupon the polyester tube shrank and tightly insulated the detector’s side surfaces. For all detector measurements, a point source of ^137^Cs was placed on the cathode side of the detectors.

## Additional Information

**How to cite this article**: Roy, U. N. *et al*. Novel ZnO:Al contacts to CdZnTe for X- and gamma-ray detectors. *Sci. Rep.* **6**, 26384; doi: 10.1038/srep26384 (2016).

## Supplementary Material

Supplementary Information
