# Supplementary material for: Novel ZnO:Al contacts to CdZnTe for X- and gamma-ray detectors
Source: Sci Rep. 2016 May 24;6:26384. doi: 10.1038/srep26384 (PMC4877641; doi:10.1038/srep26384)
Supplement: Supplementary Information [file srep26384-s1.doc]

Supplementary Information for

Novel ZnO:Al contacts to CdZnTe for X- and gamma-ray detectors

U. N. Roy^*^, R. M. Mundle, G. S. Camarda, Y. Cui, R. Gul, A. Hossain, G. Yang, A. K. Pradhan, and R. B. James

* Correspondence and requests for materials should be addressed to U.N.Roy (uroy@bnl.gov)

Thermal expansion coefficient of CdZnTe, common metals used for contacts, and ZnO are listed in Table 1. The Vickers hardness values for the common electrode metals and ZnO are also tabulated in Table 1.

**Table 1. Hardness and thermal expansion coefficient of CdZnTe and common contact metals, along with the proposed ZnO contact.**

| **Material** | **CdZnTe** | **Gold** | **Aluminum** | **Indium** | **Platinum** | **Zinc oxide** |
| --- | --- | --- | --- | --- | --- | --- |
| **Vickers hardness** |  | 216 MPa^1^ | 167 MPa^1^ | ------ | 549 MPa^1^ | 4.7 GPa^2^ |
| **Thermal expansion coefficient** | 4.8^3^  (10^-6^/ C) | 14.4^3^  (10^-6^/ C) | 23.8^3^  (10^-6^/ C) | 32.1^3^  (10^-6^/ C) | 8.9^3^  (10^-6^/ C) | ┴c 4.7^4^  ║c 2.9  (10^-6^/ K) |

**Detector configuration**

Figures 1a and 1b show a schematic of the virtual Frisch grid detectors with gold contacts on both ends and with AZO contacts on both ends, respectively. After characterization of the detector with gold contacts, the gold electrodes were polished off and then deposited with AZO contacts for comparison using the same CZT sample,


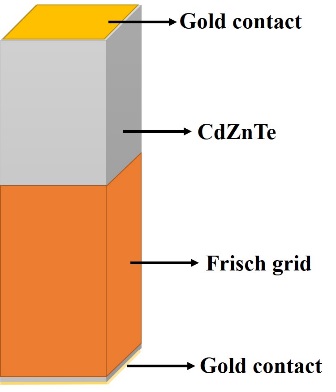

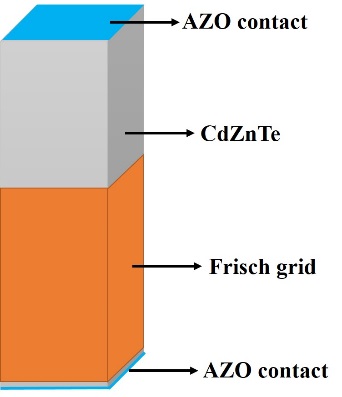


(a) (b)

Figure 1. Schematic of the virtual Frisch grid detector a) with gold contacts and b) with AZO contacts.

**Photoluminescence measurement**

We performed low-temperature photoluminescence (PL) spectroscopic analyses for the CdZnTe sample and the AZO/CZT interface. The PL spectra were acquired at ~5 K using a He-cryostat, and the samples were excited with a 488-nm laser. In order to study the CdZnTe surface and the interface and their comparison, the CdZnTe sample was polished followed by etching with 2% bromine methanol solution for two minutes. Half of the sample’s surface was masked, and the AZO was deposited on the other half. The low-temperature PL spectra for bare CdZnTe surface and the AZO-coated surface are shown in Figure 2. The inset shows a schematic of the CdZnTe surface with and without an AZO coating. The PL spectra were acquired by exciting the material with a laser on the bare CdZnTe surface and also on the AZO-coated CdZnTe surface. The features of the PL spectra are similar for the CdZnTe and AZO-coated CdZnTe surface, as shown in Figure 2. The spectra consist of a dominant


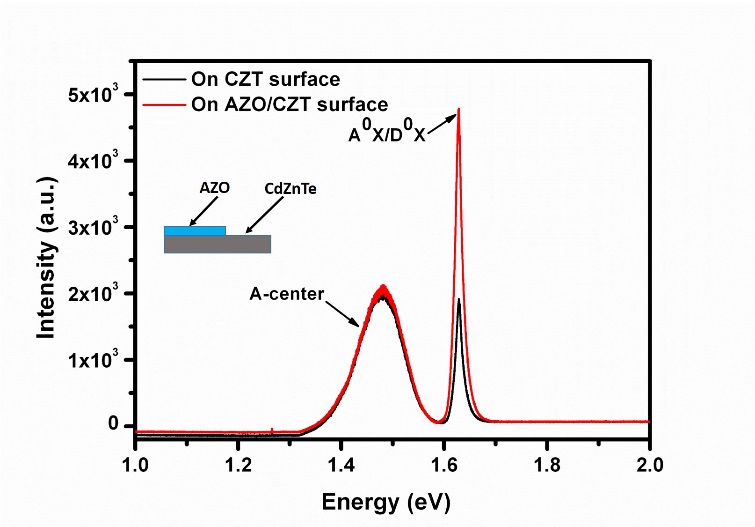


Figure 2. Photoluminescence spectra of bare CdZnTe and AZO-coated CdZnTe at ~ 5K.

peak at ~1.63 eV and a broad peak centered ~1.48 eV. The dominant peak is related to either an acceptor-bound exciton (A^0^,X) or a donor-bound exciton (D^0^,X); however, according to Schlesinger et al.^5^, the (D^0^,X) peak is typically dominant for Cd_0.9_Zn_0.1_Te. The broad peak centered at ~1.48 eV is commonly known as an A-center and is ascribed to a Cd vacancy in complex with a donor^5^. No additional peak was observed from the AZO/CdZnTe interface within the studied region from 1-2.4 eV range. As evident from Fig. 2, the ratio of the peak intensities for the (D^0^,X) and A-center is predominantly higher for the AZO/CZT interface compared to the bare CdZnTe surface. The reduction of the peak intensity for the bare CdZnTe might be due to the surface degradation, because of the time lapse from the etching to PL measurements. Thus, the AZO layer acts as a protective layer and is an indication of greater interface stability than gold layers over time, since the PL intensity of the excitonic line from the underlying layer below a gold layer was reported to decrease drastically compared to bare CdZnTe surface^6^.

**References:**

1. G. V. Samsonov, "Mechanical Properties of the Elements", [*Handbook of the physicochemical properties of the elements*](http://ihtik.lib.ru/2011.08_ihtik_nauka-tehnika/2011.08_ihtik_nauka-tehnika_3560.rar) (New York, USA, 1968).
2. I. Yonenaga, *Physica B* **308-310**, 1150 (2001).
3. W. Sang, J. Wei, Z. Qi et al., *Nucl. Instrum. Methods Phys. Res. A* **527**, 487 (2004).
4. H. Ibach, *Phys. Stat. Sol.* **33**, 257 (1969).
5. T. E. Schlesinger et al., *Materials Sc. and Engr. Reports* **32,** 103 (2001).
6. M. A. George et al., *J. Appl. Phys.* **7**, 3134 (1995).
